# A Survey of Rounding Practices in Canadian Adult Intensive Care Units

**DOI:** 10.1371/journal.pone.0145408

**Published:** 2015-12-23

**Authors:** Jessalyn K. Holodinsky, Marilynne A. Hebert, David A. Zygun, Romain Rigal, Simon Berthelot, Deborah J. Cook, Henry T. Stelfox

**Affiliations:** 1 Department of Community Health Sciences, University of Calgary, Calgary, Alberta, Canada; 2 Division of Critical Care Medicine, University of Alberta, Edmonton, Alberta, Canada; 3 Centre De Recherche, l’Université de Montréal, Montréal, Québec, Canada; 4 Département de Médecine d’Urgence, CHU de Québec, Québec City, Québec, Canada; 5 Faculty of Health Sciences, McMaster University, Hamilton, Ontario, Canada; 6 Department of Critical Care Medicine, University of Calgary and Alberta Health Services, Calgary, Alberta, Canada; Oregon Health and Science University, UNITED STATES

## Abstract

**Objective:**

To describe rounding practices in Canadian adult Intensive Care Units (ICU) and identify opportunities for improvement.

**Design:**

Mixed methods design. Cross sectional survey of Canadian Adult ICUs (n = 180) with purposefully sampled follow-up interviews (n = 7).

**Measurements and Main Results:**

Medical directors representing 111 ICUs (62%) participated in the survey. Rounding practices varied across ICUs with the majority reporting the use of interprofessional rounds (81%) that employed an open (94%) and collaborative (86%) approach, occurred at the patient’s bedside (82%), and started at a standard time (79%) and standard location (56%). Most participants reported that patients (83%) and family members (67%) were welcome to attend rounds. Approximately half of ICUs (48%) used tools to facilitate rounds. Interruptions during rounds were reported to be common (i.e., ≥1 interruption for ≥50% of patients) in 46% of ICUs. Four themes were identified from qualitative analysis of participant responses to open-ended survey questions and interviews: *multidisciplinarity*, *patient and family involvement*, *factors influencing productivity*, and *teaching and learning*.

**Conclusions:**

There is considerable variation in current rounding practices in Canadian medical/surgical ICUs. Opportunities exist to improve ICU rounds including ensuring the engagement of essential participants, clearly defining participant roles, establishing a standardized approach to the rounding process, minimizing interruptions, modifying the role of teaching, utilizing a structured rounding tool, and developing a metric for measuring rounding quality.

## Introduction

Effective communication among healthcare providers (subsequently referred to as providers) is essential for high quality patient care. Ineffective communication is associated with medical errors and adverse events[1–3]. For patients admitted to the Intensive Care Unit (ICU), the most important regularly scheduled communication occurs during patient care rounds—a key forum for providers to review the patient’s condition and progress, discuss diagnostic, preventative, and therapeutic options, and make important patient care decisions.

A systematic review performed by Lane et al (2013)[4], which was recently updated[5], identified 13 best practices for ICU patient care rounds. These included implementing interprofessional rounds (physician, nurse, and pharmacist at minimum)[6–15]; standardizing practices[16–19]; defining roles for all participants[20–23]; using a structured tool[24–35]; reducing time spent on non-essential activities[17,18]; minimizing interruptions[36–38]; developing and documenting daily goals[22,31,39,40]; considering the best location of rounds (bedside vs. conference room) to optimize patient-centeredness and efficiency[36,41–43]; and establishing both an open and collaborative discussion environment[16,42–44]. All of these best practices were reported to potentially improve rounds either by increasing provider satisfaction, reducing rounding time, or improving patient outcomes. A separate systematic review of pediatric ICUs[45] and related literature [46–55] reported that family presence and participation in rounds created a patient-centered environment, enhanced communication, and increased both family and provider satisfaction. The role of families in rounds in adult ICUs is less well defined and two recent studies have reported both positive and negative provider perceptions[56,57].

While best rounding practices[4] have been proposed, it is unknown if these practices are used in daily patient care. We therefore conducted a cross sectional survey of Canadian adult medical/surgical ICUs with follow up interviews to describe current rounding practices and to identify opportunities for improvement.

## Materials and Methods

This study was approved by the Conjoint Health Research Ethics Board at the University of Calgary, which waived the need for written informed consent.

### Study Design

This was a two-part mixed methods study. First, we surveyed ICU medical directors to describe the structure, process, and outcomes of rounds in their adult ICUs. Second, we performed follow up interviews to further characterize rounding practices.

### Survey and Interview Guide Development

We employed a conceptual framework derived from the Institute of Medicine’s Aims for 21^st^ Century Healthcare[58], and Donabedian’s quality of care framework[59].We developed survey questions using the 9 best practices previously reported in the literature that were most amenable to survey/interview data collection: interprofessional rounds, standardizing time and location, defining participant roles, tool use, minimizing interruptions, focusing on goal development, promoting patient-centeredness through bedside discussion, promoting efficiency by using conference room discussion, and ensuring an open and collaborative environment [4]. Questions were developed to assess self-perceived quality and room for improvement of patient care rounds using an ordinal scale from 1 to 10. We developed open-ended style questions to assess participant’s opinions on positive and negative aspects of rounds. Participants were asked to forward (email, fax, mail) any tools used to facilitate rounds. We pilot tested the survey to assess length, flow, readability, and clinical sensibility using a convenience sample of 20 ICU providers (S1 File). One of the authors (S.B.) a physician fluent in both English and French translated the survey into French and translated responses to open text questions from French into English for analysis.

We developed interview questions to elicit individual provider roles during rounds, tools used during rounds, facilitators and barriers to rounding practices, and suggestions to improve rounding practices. The interview guide was pilot tested on a convenience sample of ICU physicians (S2 File).

### Data Collection

We surveyed Medical Directors of ICUs (or their designates) that satisfied the following inclusion criteria: 1) located in Canada; 2) primarily adult patients (≥18 years old); 3) population of medical and/or surgical patients. Specialty ICUs (including but not limited to cardiac/cardiovascular, neurologic, and burn ICUs) and pediatric ICUs were excluded from the study as their highly specific patient populations may influence their rounding practices. We identified medical directors by searching hospital websites, phoning hospitals, and consulting provincial ICU leaders. Medical directors were sent the survey using a secure online survey software (www.surveymonkey.net). Non-respondents were sent up to three reminders at two-week intervals.

We conducted semi-structured individual telephone interviews with ICU medical directors. Participants were purposively sampled (ensuring representation of both small and large ICUs and academic and non-academic ICUs from diverse geographic regions) to maximize the diversity of perspectives and experiences captured. The interviews were audio taped and transcribed for analysis. Interviews continued until saturation was reached.

### Data Analyses

We used descriptive statistics to report ICU rounding practices. Categorical variables were summarized as counts and percentages; continuous variables were reported as means and standard deviations (SD). Content items from collected tools were also analyzed descriptively.

Ordinal logistic regression was used to identify predictors of both self-rated rounding quality and self-rated room for improvement. The quality scores were combined into five groups (1–2, 3–4, 5–6, 7–8, 9–10) for analysis. Potential predictors were organized into three groups (according to Donabedian’s structure, process, outcome framework[59]) and each of these groupings was analyzed in a separate model. All models were created using backward selection, where all predictors were initially included in the model and predictors with a p-value < 0.10 were sequentially eliminated[60].

An inductive thematic analysis was performed jointly by two authors (J.K.H. & M.A.H.) on the open-ended survey questions and interview transcripts[61]. Data from the open-ended questions and interview transcripts were extracted and numbered by participant. The two authors iteratively analyzed the data by identifying common themes and subthemes and organizing the data respectively until both agreed on the themes, subthemes, their definitions, and the appropriate sorting of all comments.

## Results

From 180 eligible ICUs that were invited to participate in the survey, 107 individuals representing 111 ICUs (62%) from nine of ten provinces completed the survey; most (94%) respondents answered the open-ended questions. Using purposive sampling we contacted 30 individuals for follow-up interviews; 10 responded to our invitation to participate and 7 interviews were performed. The characteristics of the ICUs are summarized in Table 1; responses to all survey questions are included in Table A in S3 File.

**Table 1:** Characteristics of Participating ICUs.

Characteristic		Number of ICUs	
		Survey Participants (N = 111)	Interview Participants (N = 7)
Province	Alberta	13	2
	British Columbia	21	1
	Manitoba	4	-
	New Brunswick	3	-
	Newfoundland	1	1
	Nova Scotia	2	-
	Ontario	32	3
	Prince Edward Island	0	-
	Quebec	29	-
	Saskatchewan	6	-
Number of Beds	< 10	36	1
	10–19	42	3
	20–29	25	3
	30–39	7	-
	Missing	1	-
Model of Care	Open: Intensivist Consult	15	-
	Closed: Intensivist Directed	94	7
	Mixed	2	-
Academic ICU	Yes	79	5
	No	32	2
Participants Role	Medical Director	45	4
	Intensive Care Physician	49	3
	Nurse Manager	12	-
	Patient Care Coordinator	1	-
Types of Patients^a^	Medical	110	7
	Surgical	108	7
	Cardiac—Medical	61	3
	Neurologic	56	4
	Trauma	44	3
	Cardiac–Surgical	20	2
	Burns	14	1

^a^ Numbers sum to greater than 111 (survey) or 7 (interviews) as some ICUs cared for multiple different types of patients

Four themes (multiple subthemes) emerged from analysis of the open-ended survey and interview questions: multidisciplinarity, patient and family involvement, factors influencing productivity, and opportunities for teaching and learning (Table 2). Self-reported rounding practices are summarized in Table 3. Responses to closed and open-ended survey and interview questions are reported below within the relevant themes and subthemes reflected in participant quotes.

**Table 2:** Thematic Analyses of Open-Ended Survey and Interview Questions.

Themes	Subthemes	Items	Category of Care*
1. Role of Interprofessionalism	1.1 Interprofessional Team		Structure
	1.2 Interaction		Structure
	1.3 Open and Collaborative Environment		Structure
	1.4 Team Environment		Structure
	1.5 Communication	1.5.1 Within ICU Care Team	Structure
		1.5.2 Outside ICU Care Team	
	1.6 Leadership and Roles		Structure/Process
2. Patient and Family Involvement	2.1 Family		Process
	2.2 Patient		Process
	2.3 Both Patient and Family		Process
3. Factors Influencing Productivity	3.1 Interruptions	3.1.1 Pages/Phone Calls	Process
		3.1.2 Consultations	
		3.1.3 Disruptive Behaviour	
		3.1.4 Needs of Other Patients	
		3.1.5 Non-Specific Causes	
	3.2 Timing	3.2.1 Timely	Structure
		3.2.2 Too Long	
	3.3 Inconsistent Attendance		Structure
	3.4 Inefficiencies		Structure
	3.5 Inconsistent Rounding Practice		Structure
	3.6 Care Plan Created		Process
	3.7 Tools to Facilitate Rounds		Structure
4. Opportunities for Teaching and Learning	4.1 Professional		Process
	4.2 Content		Process

*Categorized according to the Donabedian Structure, Process, and Outcome model of care. Structure refers to characteristics of the setting in which care occurs, this includes material resources, human resources, and organizational structure. Process refers to the actual giving and receiving of care including both the patient seeking out care and the providers activities in making diagnosis and treatment decisions. Outcome refers to the effects of care on the patient and/or population including the patient’s satisfaction with care[59].

**Table 3:** Rounding Practices.

Practice Item		Reported Frequency by Intensive Care UnitN = 111 [n (% of ICUs)]
Open Environment		104 (94%)
Collaborative Environment		95 (86%)
Interprofessional		90 (81%)
Standard Start Time		88 (79%)
Standard Start Location		62 (56%)
Rounding Tool Use		53 (48%)
Location of Rounds	Patient’s Bedside	91 (82%)
	Conference Room	13 (12%)
	Combination of Both	7 (6%)

### Role of Interprofessonalism

#### Interprofessional Team

The composition of the interprofessional rounding team varied across ICUs. The majority of ICUs reported that attending physicians (98%), bedside nurses (94%), respiratory therapists (89%), and pharmacists (85%) regularly attended rounds. Nine other providers were also reported to attend rounds by some ICUs (Figure A in S4 File). Ninety ICUs (81%) reported rounds that were interprofessional according to Lane et al.’s definition (included at minimum an attending physician, bedside nurse, and pharmacist) [4] (Table 3). Survey respondents indicated that a positive aspect of the interprofessional team was that it improves the safety and quality of care, specifically: “overlapping responsibility hopefully results in catching ‘near misses’ or mistakes” and “[interprofessional rounds] lead to the best outcomes and shortest length of stay for the patient”. All participants reported their rounds were usually or always safe, and that medical errors never or sometimes occurred as a result of rounding practices (Figure B in S4 File). Conversely, survey respondents noted that negative aspects of a interprofessional team include: “some of the team members will go several patients without having meaningful contributions” and “because there are so many people it can be challenging to maintain a focused conversation.”

#### Interaction

Provider interaction was cited as one of the benefits to interprofessional rounds, especially: “the ability for all to participate” and “new ideas arising because of the process of interacting.” Other survey respondents cited that the different providers worked well together. Negative interactions were also reported including, “rounds vary depending on the individual intensivist as each interacts differently with the team.”

#### Open and Collaborative Team Environment

ICUs reported having an open (94%) and collaborative (86%) environment during rounds (Table 3). Survey respondents cited an open environment where “everyone has a voice” as a positive aspect of rounds. The commitment of the team members and being able to work together as a team were cited as a positive aspect of rounds.

“We do morning rounds sitting down around a conference table, it puts everyone on more or less a level playing field. So everyone can be seen and everyone has a place at the table so to speak and everyone can bring their issues.”

#### Communication

Communication between the different ICU providers, including having different providers together for “communication of the plan of care and advancement of care” was cited as a positive aspect of rounds. Communication was also brought up as a negative aspect of rounds in reference to a lack of dialogue between ICU and non-ICU providers (e.g., consulting physicians and surgeons).

“[The worst parts of rounds is] the lack of communication with the [non-ICU] teams and trying to guess what they are thinking while on our own ICU rounds”

#### Leadership and Roles

Participants reported different perspectives on provider roles in rounds. Physicians (attending and/or fellow) and nurses were both identified as potential leaders of rounds. Other providers were reported to be involved in presenting information and contributing to the development of a plan. One participant reported that respiratory therapists only participated on rounds for the mechanically ventilated patients.

“People who are participating know their roles and what they are expected to contribute and know also what to expect from others.”

### Patient and Family Involvement

Intensive care units reported that awake and aware patients (83%) and patient’s families (66%) participated in rounds. Five roles were described for the patients or their families among the ICUs that included them in rounds (Table A in S4 File). The majority of interview participants listed the patient as a passive participant in rounds who was there to listen and receive clarification. Family members were more frequently identified as active participants who could provide information about the patient’s baseline functional and medical status, express wishes and provide input into decision-making.

“The involvement of patients and families is evolving and becoming more consistent.”

The majority (73%) of comments suggested that patient and family involvement in rounds was positive. Having the family present to discuss the care plan and treatment options was identified as a positive aspect of rounds for most, but a negative aspect of rounds for a minority (26%) of respondents. Family presence was specifically noted as being negative if “families [were not] clear about goals of care.”

The location of rounds was reported to directly affect patient and family participation. A small minority (12%) of ICUs conducted rounds away from the bedside in a conference room (Table 3).

“Rounds are conducted in a conference room. The patients and families are not part of rounds for this reason.”

### Factors Influencing the Productivity of Rounds

#### Interruptions

**Fig 1:**
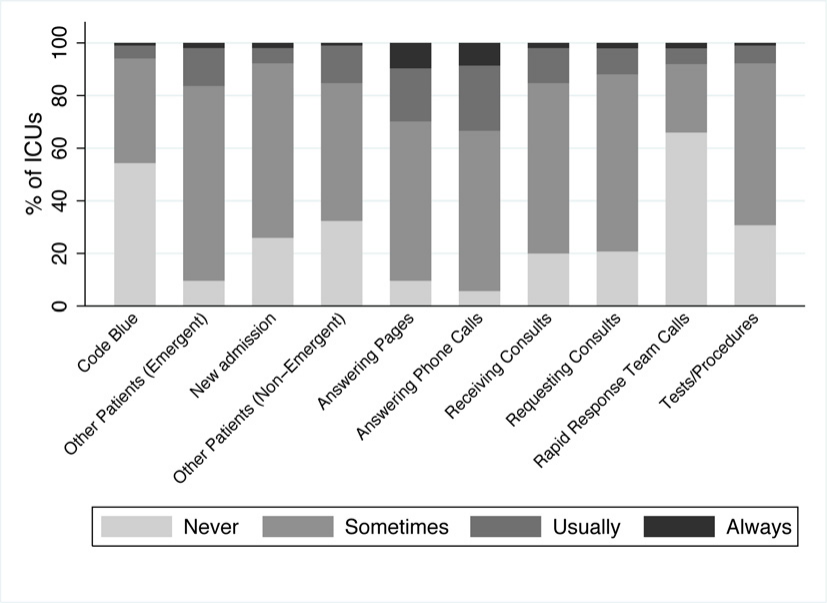
Sources of Interruption During Patient Care Rounds.

Forty-six percent of survey respondents reported that rounds were usually or always interrupted (Fig 1). The most common interruptions reported were answering pages and phone calls. Two distinct types of interruptions were described: those initiated by persons outside the rounding team (e.g. pages, phone calls, consultations, and requests to address the needs of other patients) and those generated by members of the rounding team (e.g. side conversations and team members leaving to perform other tasks). Participants from ICUs where rounds were performed at the patient’s bedside reported that rounds were usually or always interrupted in a similar proportion to those where rounds were conducted in a conference room (50% vs. 33%, p = 0.11). Interruptions were the most common issue reported to affect productivity within the survey responses.

“[The worst aspects of rounds are] interruptions, phone calls, and consults. [Also] delaying rounds on a patient because there is another patient that needs more urgent attention.”

#### Timing

The timing and duration of rounds was reported to impact quality. Survey respondents indicated that a median of 11 patients [interquartile range (IQR) 8 to 14 patients] were seen on rounds per day, with a median of 15 minutes (IQR 10 to 20 minutes) spent with each patient. Across all units the total median reported rounding time was 168 minutes (IQR 102 minutes to 245 minutes). The majority of ICUs (79%) specified a standard start time for rounds (Table 3). Survey respondents and interview participants noted that establishing a time for a specific patient’s rounds as a challenge and frustration. Survey respondents noted that lengthy rounds negatively affected productivity, contributed to health care provider fatigue, inattention, and information being missed during the round and delayed patient care, especially transfers into and out of the ICU.

“Lengthy rounds create difficulty maintaining team focus, cause fatigue as rounds ‘drag’ on, and a large volume of information is managed creating the risk of missing important information”

#### Inconsistent Attendance

Inconsistent attendance of providers, including physicians, nurses, respiratory therapists, pharmacists, and social workers was reported to negatively affect productivity during patient care rounds. Interview participants identified increasing provider attendance as a strategy for improving rounds.

“Sometimes the person on call for the [previous] night won’t attend rounds” “Unavailability of the beside nurse” “Social work doesn’t attend rounds”

#### Inefficiencies

The majority of survey respondents reported having efficient rounds (79%) that started at a standard location (56%) and were timely (95%) and equitable (ensuring each patient received the time and attention they required) (88%) for patients (Figure B in S4 File). Inefficiencies identified by survey respondents and interview participants included: lack of standardized rounding order, coordinating tests and procedures during rounds, spending too much time on minor issues, and spending too much time tracking down information and writing notes.

“At times, discussions do get beyond patient care in rounds which is essentially not needed”

#### Inconsistent Rounding Practices

Inconsistencies in practice were reported to adversely affect rounds. These included variation in rounding structure and process according to the physician leading the rounds and the organization and experience of the providers presenting during rounds.

“The worst aspect of rounds is inconsistent physician practice (nursing practice as well)”

#### Care Plan Created

Survey respondents and interview participants highlighted that the primary purpose of rounds was to create a care plan that was understood by all members of the health care team. Nearly all ICUs (89%) reported that rounds usually or always resulted in a tangible care plan for the patient (effectiveness, Figure B in S4 File).

“[The best aspect of rounds is] actions are clearly identified for the next 24-hour period.”

#### Tools to Facilitate Rounds

About half of ICUs (n = 49, 48%) reported using a tool to facilitate rounds. Among these 31 reported using a checklist, 16 a goal sheet, and 17 another type of tool. Fifteen ICUs reported using more than one tool to facilitate patient care rounds. Fourteen ICUs provided copies of their tool(s), which were classified as checklists (a list of discussion points) or worksheets (containing items to be filled in) (Table B in S4 File). The interview participants mostly cited tool use as being positive and beneficial for both the patients and providers, although it was reported that tool use sometimes increased pre rounding preparation time.

“[The checklist] works pretty well because it’s not uncommon that we find something that we haven’t discussed yet. You know, had we not [used the checklist] we might have passed over something that was important.”

### Teaching and Learning

Survey respondents reported that a median of 80% (IQR 70–87.5%) of rounding time was spent on patient care activities and a median of 20% (IQR 10–25%) on teaching. The opportunity for teaching and learning for all health care providers was reported as positive and respondents from academic ICUs indicated that this was one of the purposes of rounds. Negative aspects included disruptions (e.g., trainees coming in and out of rounds for education purposes) associated with teaching as well as unequal time spent with patients.

“Sometimes teaching causes more time to be spent at one bedside and it is hard to say if this is justified.”

### Self-Reported Rounding Quality and Room for Improvement

**Fig 2:**
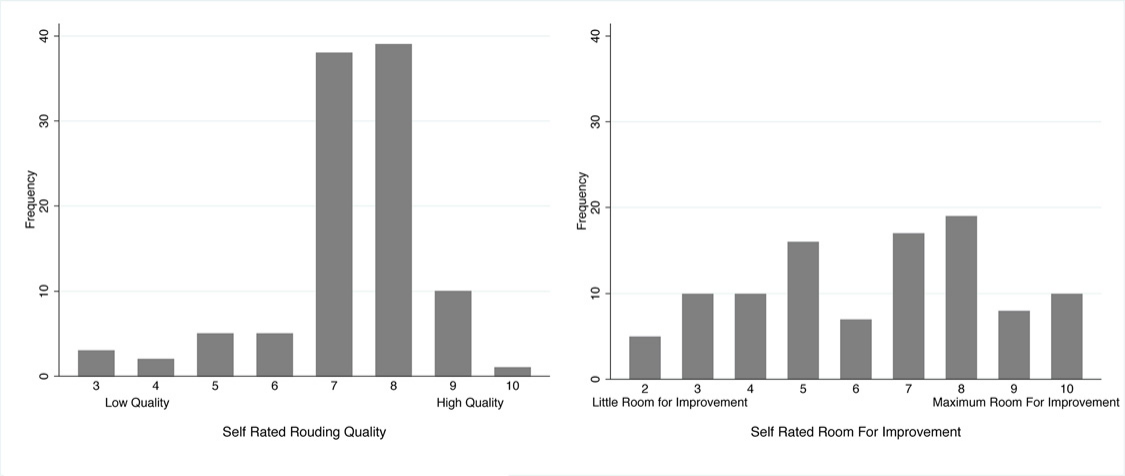
Self-Rated Rounding Quality and Room For Improvement.

Participants from each ICU were asked to provide a rating of their perceived rounding quality and room for improvement in rounds on an ordinal scale from one through ten (Fig 2). The median scores for perceived quality and room for improvement were 7 (interquartile range 7–8) and 7 (interquartile range 5–8) respectively. There was no association between participants self reported quality and room for improvement score (p = 0.2380) (Figure C in S4 File).

Table 4 summarizes the multivariable adjusted ordinal regression analyses of ICU rounding structures, processes and outcomes associated with self-reported perceptions of rounding quality and opportunities for improvement. Frequent interruptions were associated with significantly decreased perceptions of rounding quality and increased perceived room for improvement. Conversely, timely rounds were associated with perceptions higher of rounding quality and a decreased need for improvement.

**Table 4:** Rounding Variables Associated with Self-Reported Rounding Quality and Room for Improvement.

Regression Model	Category	Variable	Adjusted Odds Ratio (95% CI)^[^ ^a^ ^]^	P–Value
Self Reported Rounding Quality	Structure	Academic Institution	0.417 (0.148–1.172)	0.097
		Standard Start Time	10.646 (2.707–41.869)	0.001
	Process	Frequent Interruptions^[^ ^b^ ^]^	0.321 (0.121–0.853)	0.023
	Outcome	Safe	2.537 (0.972–6.619)	0.057
		Timely (Not delaying patient care)	4.806 (1.507–15.326)	0.008
Self Reported Room for Improvement	Structure	Time Spent Per Patient (minutes)^[^ ^c^ ^]^	1.062 (1.015–1.111)	0.009
	Process	Frequent Interruptions^[^ ^b^ ^]^	2.728 (1.318–5.648)	0.007
	Outcome	Safe	0.306 (0.145–0.648)	0.002
		Timely (Not delaying patient care)	0.434 (0.180–1.048)	0.063

^a^ Odds ratio > 1 for self reported rounding quality indicates the variable is positively associated with an increased odds of reporting a higher rounding quality score on the ordinal scale. Odds ratio > 1 for self reported room for improvement indicates the variable is positively associated with an increased odds of reporting more room for improvement score on the ordinal scale.

^b^ Reporting at least one interruption usually or always occurs during rounds

^c^ Included as continuous variables

## Discussion

### Findings

We used mixed methods to describe rounding practices in Canadian ICUs. Survey respondents and interview participants highlighted four key themes: multidisciplinarity, patient and family involvement, factors influencing productivity, and opportunities for teaching and learning. We identified variable application of the practices proposed in the systematic review by Lane et al. [4]; some practices (open, collaborative, and interprofessional environment) were widely reported while others (tool use, standard start location) less so. Interruptions were reported to increase rounding time and decrease communication quality and rounding efficiency. Overall, ICUs reported high rounding quality, but identified opportunities for improvement.

### Recommendations

**Fig 3:**
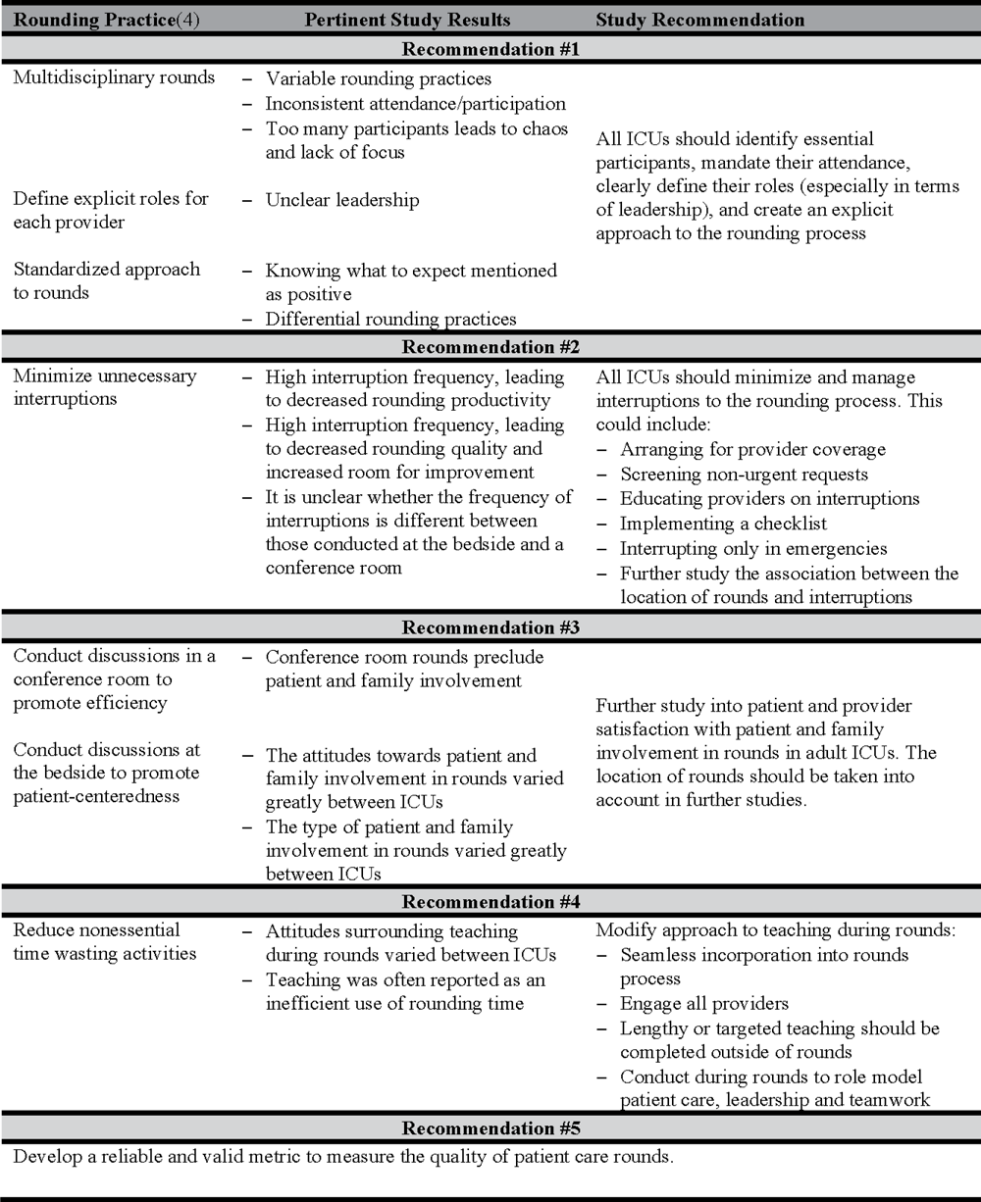
Study Recommendations.

Our study highlights five opportunities for improving rounds (Fig 3).

The first recommendation from this study is that ICUs identify essential rounds participants relevant for their individual patient populations, mandate their attendance, define explicit roles for each participant, and create an explicit approach to the rounding process. The negative aspects of multidisciplinarity suggest that the roles of the interprofessional team members and the rounding process may be unclear. Some participants reported a lack of consensus on who was the most appropriate leader for rounds and important variation in the structure and process of rounds among different physicians. Some providers did not consistently participate in or even attend rounds. Communication with non-ICU providers was reported as being poor or non-existent indicating that the role of the non-ICU provider during rounds might be unclear. This suggests that rounds should have a standardized structure and process, include a team of providers with clearly delineated roles and be directed by a provider (regardless of profession) with strong communication and team management skills. While encouraging the participation of interprofessional providers in rounds can be justified from the existing evidence-base, the optimal organizational structure and process for the team is less clear, would benefit from additional research and could include elements of leadership and teamwork training.

The second study recommendation is to minimize and manage interruptions during rounds. The number and variety of interruptions suggest that a multifaceted approach might be necessary to solve this problem. One option is to arrange for provider coverage during rounds (rapid response team, or covering MD/RN) to handle new urgent requests (e.g., patients not currently under the ICU team’s care). Non-urgent requests, pages, and phone calls could be screened, triaged and collated by a provider not participating in rounds (e.g., one resident or designated nurse) and presented between patients or after rounds depending on time sensitivity. An education program regarding the potential risks associated with interrupting rounds (errors of omission or commission due to distractions, and delayed task completion) may help reduce non-urgent interruption frequency. In conjunction with this education program, an “interrupt only in emergencies” contract could be introduced in the unit to change the unit culture surrounding interruptions. A rounding tool could be used as a safeguard against missed information due to interruptions.

The third study recommendation is that additional research is needed to determine the role of patients and families in rounds. Our study findings showed that the involvement of the patient and family in rounds varied across ICUs. When the patient/family was involved they could take on a range of roles from observation to involvement in shared decision-making. While patient and family participation has been reportedly positive in neonatal and pediatric ICUs, there is limited research to support this is adult ICUs and further study is warranted[45,56,57].

The fourth study recommendation is that teaching be seamlessly incorporated into rounds so that it does not distract from patient care (but rather enhances care) and does not appreciably lengthen rounds or induce provider fatigue. While teaching and learning are essential elements of practice, they may also be seen as a hindrance to the rounding process, especially if teaching and learning are not viewed as the purpose of patient care rounds by some participants. Teaching during rounds should engage all providers as best possible, and may involve a range of strategies such as eliciting suggested approaches to a problem, listing possible complications, briefly citing the evidentiary basis for decisions and other role modeling. Supplemented by profession-specific (e.g. residents) sessions outside of rounds to ensure an inclusive and streamlined process. The ideal balance between concrete patient care activities and educating providers is unknown and likely to vary between circumstances (e.g., less teaching and more patient care when there are urgent patient care activities). Additional research would be valuable in delineating the frequency (i.e., what proportion of patients), duration (i.e., what is the optimal length of time), organization (i.e., how many teaching points per education episode), content (i.e., how is the teaching linked to patient care decision-making) and participants (i.e., which providers should be engaged) in rounding related educational activities and how these are impacted by and on patient care activities (i.e., what is the interaction between patient care activities and educational activities).

The final recommendation is the need to develop a comprehensive measure to evaluate ICU rounding practice quality. Overall, ICUs reported high rounding quality, but then identified many opportunities for improvement. How can these conflicting reports be reconciled? Medical directors may perceive continuous quality improvement as important regardless of the care provided. This perspective would be consistent with the observed absence of a relationship between self-reported rounding quality and perceived room for improvement (Figure C in S4 File). Alternatively, self-report may be a poor indicator of rounding quality. For example, the factors associated with self-reported quality of rounds and room for improvement in the quantitative data included timeliness, interruptions, and safety whereas multidisciplinarity, team environments, and communication were highlighted as being some of the best and worst aspects of rounds in the qualitative data. These discrepancies could represent different constructs for improvement identified using different methodologies, and/or reflect the challenges of using self-report as a metric of the quality rounding. We based our survey and interview guide on a systematic review of evidence informed ICU rounding practices, but we are only aware of one validated measure of rounding quality[62]. This metric focuses on the content discussed during rounds and interdisciplinary collaboration, but does not assess other aspects of rounds identified as areas for potential improvement. Without reliable and valid metrics it is difficult to objectively assess rounding quality, identify gaps and evaluate the effect the efficacy of interventions designed to improve rounding quality.

Finally, we have proposed a comprehensive rounding tool (S5 File) to be used in conjunction with these recommendations. While the use of tools to facilitate rounds has been previously recommended[4] and using a tool was often cited as a positive aspect of rounds in our survey data, only 48% of ICUs reported using rounding tools. Why do less than half of ICUs report using a rounding tool? One potential explanation is that developing a locally customized tool is time consuming and there are currently no recommendations on what items a rounding tool should contain. A generic tool may address this challenge, but risks not meeting local needs. The tool proposed in our manuscript represents a composite of items identified from the tools collected from ICUs in the study, interview responses and recommendations from checklist creation guidelines[63]. It represents one potential starting point for ICUs interested in adopting and/or adapting an ICU rounding tool, but like all instruments will need to be evaluated for both intended (e.g., increasing the effectiveness of rounds) and unintended (e.g., increasing the duration of rounds) consequences.

### Limitations

These results are generalizable to small and medium sized academic medical and/or surgical ICUs utilizing a closed model of care, but it is unclear whether they reflect the rounding methods or experiences of subspecialty ICUs or ICUs using different models of care. Furthermore, these results represent the perspectives of ICU medical directors or their designates, the majority of whom are physicians; other providers may have different perspectives and these should be considered in future research and quality improvement initiatives. The data are self-reported, may represent idealized views, and may therefore underestimate opportunities to improve the quality of rounds. Finally, the observations are largely reported as independent measures for the purposes of simplicity, but may be interrelated. For example, incorporating interprofessional rounds with clearly defined participant roles may increase provider satisfaction and decrease rounding variability, but may also impact the duration of rounds.

## Conclusions

This study found that the conduct of ICU rounds is a crucial but variable aspect of patient care, with differential adoption of recommended practices. Opportunities to improve rounds include establishing a standardizing team composition and roles, minimizing interruptions, determining the optimal location(s) for rounds, seamlessly integrating education with patient care, and incorporating a structured tool to facilitate rounds. There is an urgent need to develop instruments to objectively measure rounding quality to guide quality improvement efforts.

## Supporting Information

S1 File National Survey of ICU Patient Care Rounds. (PDF)

S2 File Interview Guide. (PDF)

S3 File Survey Responses. Table A. Survey Responses.
(PDF)

S4 File Figures and Tables. Figure A. Health Care Providers Who Regularly Attend Patient Care Rounds. Figure B. Evaluation of Rounds Using the IOM Aims for 21^st^ Century Health Care[58]. Figure C. Relationship Between Self-Reported Rounding Quality and Room For Improvement Scores. Table A: Role of the Patient and Family During Rounds. Table B: Characteristics of Tools Provided.
(PDF)

S5 File Rounding Tool. (PDF)
