# Supplementary material for: A Survey of Rounding Practices in Canadian Adult Intensive Care Units
Source: PLoS One. 2015 Dec 23;10(12):e0145408. doi: 10.1371/journal.pone.0145408 (PMC4689549; doi:10.1371/journal.pone.0145408)
Supplement: S1 File — (PDF) [file pone.0145408.s001.pdf]

National Survey of ICU Patient Care Rounds

National Survey of ICU Patient Care Rounds

Dear Intensive Care Unit Health Care Provider,

Attached is a short survey designed to learn what current patient care rounding practices are across Canadian adult Intensive Care Units. You are being contacted as you have extensive experience in intensive care. This survey will take less than ten minutes to complete and your input is valuable to this research project.

The aim of this research project is to outline current patient care rounding practices in Canadian adult ICUs. We are hoping to gain information on what current rounding practices are and what various health care providers think are the best and worst aspects of patient care rounds.

Your participation in this survey is voluntary and all results will be kept strictly confidential. This survey has been approved through the Conjoint Health Research Ethics Board at the University of Calgary; your participation in this survey implies consent.

If you have any questions or concerns please contact Jessalyn Holodinsky (jkholodi@ucalgary.ca, 403-944-0746).

Thank you for your participation.

National Survey of ICU Patient Care Rounds

Demographic Information

1. What is your role in the Intensive Care Unit?

- ☐ Medical Director/Head Intensivist
- ☐ Unit Manager
- ☐ Attending Physician
- ☐ Clinical Nurse Educator

Other (please specify)

2. What is the name of the hospital where your ICU is located?

3. What province is your hospital located in?

4. What types of patients are usually cared for in your ICU? (check all that apply)

- ☐ Medical
- ☐ Surgical
- ☐ Trauma
- ☐ Neurologic
- ☐ Cardiac/Cardiovascular Medical
- ☐ Cardiac/Cardiovascular Surgical
- ☐ Burns
- ☐ Pediatric

Other (please specify)

5. Can your ICU care for ventilated patients?

- ◯ Yes
- ◯ No

6. On average, how many operational beds are in your ICU?

7. Does your ICU use an open or closed model of care?

- ◯ Open: consult physicians direct care of their individual patients with the assistance of ICU staff
- ◯ Closed: patient care is directed by ICU physicians

Other (please specify)

8. Does your ICU regularly host residents?

- ◯ Yes
- ◯ No

Rounding Practices

9. Which of the following types of rounds happen on a daily basis in your unit? (check all that apply)

- ☐ Pre-rounding (any rounding activities done before formal patient care rounds)
- ☐ Multidisciplinary rounds
- ☐ Post-rounding (any rounding activities done after formal patient care rounds)
- ☐ Sign-out rounds (reviewing patients before handing over responsibility to a covering physician)
- ☐ Diagnostic Imaging rounds (rounds dedicated to patient imaging, ex: reviewing x-rays)
- ☐ Rapid fire or bullet rounds (physician meeting with RT/charge RN before rounds to quickly discuss extubation/discharge decisions)

Other (please specify)

Multidisciplinary Rounding Practices

The rest of this survey pertains to multidisciplinary patient care rounds. Here we define this as the scheduled discussion of individual patients to review clinical information and develop care plans. You will be asked to answer questions regarding the structure and process of patient care rounds in the ICU in which you work. Should your ICU care for both pediatric and adult patients please respond to these questions considering patient care rounds on adult patients only.

10. In your unit who is involved in patient care rounds on a regular basis? (check all that apply)

- [ ] Attending Physicians
- [ ] ICU Fellows
- [ ] ICU Residents
- [ ] Medical Students (clinical clerks, medical students, etc.)
- [ ] Bedside Nurses
- [ ] Other Nursing Positions (nurse clinicians, charge nurses, nurse educators, etc.)
- [ ] Nurse Practitioners
- [ ] Pharmacists
- [ ] Respiratory Therapists
- [ ] Dieticians/Nutrition Services
- [ ] Physiotherapists
- [ ] Social Work

Other (please specify)

11. In your unit where do patient care rounds regularly occur? (check all that apply)

- [ ] At the patients bedside
- [ ] In the hallway next to the patients room
- [ ] In a conference room

Other (please specify)

Multidisciplinary Rounding Practices

The following questions ask how often particular events happen during patient care rounds in your ICU. Please answer how often these items happen during a typical week of patient care rounds in your unit.

12. Is there a standard starting time for patient care rounds in your unit?

- ◯ Rarely or Never (0 days per week)
- ◯ Sometimes (1-3 days per week)
- ◯ Usually (4-6 days per week)
- ◯ Always (7 days per week)

13. Is there a standard starting location for patient care rounds in your unit?

- ◯ Rarely or Never (0 days per week)
- ◯ Sometimes (1-3 days per week)
- ◯ Usually (4-6 days per week)
- ◯ Always (7 days per week)

14. Are patient care rounds an open environment in your unit where all participants feel safe to voice their opinions?

- ◯ Rarely or Never
- ◯ Sometimes (< ½ of all patient rounded on)
- ◯ Usually (> ½ of all patients rounded on)
- ◯ Always

15. Are patient care rounds a collaborative environment in your unit where all health care providers are encouraged to participate and all opinions are considered when creating the patient care plan?

- ◯ Rarely or Never
- ◯ Sometimes (< ½ of all patients rounded on)
- ◯ Usually (> ½ of all patients rounded on)
- ◯ Always

16. How often are patient care rounds interrupted by the following events?

	Rarely or Never	Sometimes (< ½ of all patients rounded on)	Usually (> ½ of all patients rounded on)	Always
Answering pages	○	○	○	○
Answering phone calls	○	○	○	○
Requesting consultations	○	○	○	○
Receiving consultations	○	○	○	○
Emergent issues concerning other patients	○	○	○	○
Non-emergent issues concerning other patients	○	○	○	○
Code blue	○	○	○	○
Rapid response team/Medical emergency team/Outreach team calls	○	○	○	○
New patient admissions	○	○	○	○
Tests/Procedures	○	○	○	○

Other (please specify)

Patient Involvement In Rounds

The following questions are about the patient being involved in the patient care rounding process. When answering these questions please consider only patients who are awake and aware of their hospitalization.

17. If the patient is awake and aware, how is the patient handled during patient care rounds in your unit?

- ◯ The patient is encouraged to participate and it is preferable that they participate
- ◯ The patient is allowed to participate but are not specifically invited or encouraged
- ◯ The patient is allowed to participate but it is preferable that they receive updates outside of patient care rounds
- ◯ The patient is not allowed to attend rounds

Patient Involvement In Rounds

18. If the patient is to participate in rounds what is their role? (check all that apply)

- [ ] Observe
- [ ] Provide additional information about them self
- [ ] Ask questions
- [ ] Participate in shared decision making
- [ ] Receive an update

Other (please specify)

Family Involvement in Rounds

The following are questions are about the role of the family of the patient in the patient care rounding process.

19. How is the patient's family handled during patient care rounds in your unit?

◯ The family of the patient is encouraged to attend rounds and it is preferable that they attend

◯ The family of the patient is allowed to attend rounds but are not specifically invited or encouraged

◯ The family of the patient is allowed to attend rounds but it is preferable that they receive updates outside of patient care rounds

◯ The family of the patient is not allowed to attend rounds

Family Involvement in Rounds

20. If the family of the patient is to attend rounds, what is their role? (check all that apply)

- [ ] Observe
- [ ] Provide additional information about the patient
- [ ] Ask questions
- [ ] Participate in shared decision making
- [ ] Receive an update

Other (please specify)

21. How often does the typical family of the patient attend patient care rounds?

- ◯ Rarely or Never (0 days per week)
- ◯ Sometimes (1-3 days per week)
- ◯ Usually (4-6 days per week)
- ◯ Always (7 days per week)

Multidisciplinary Rounding Practices

22. How many patients are typically rounded on in one day?

23. How much time (in minutes) is typically spent with each patient?

24. What percentage of patient care rounds is spent on the following items:

Patient Care

Teaching

Other

Rounding Instruments

25. Does your unit use any of the following during patient care rounds? (check all that apply)

- ☐ A rounding checklist
- ☐ A patient goal sheet
- ☐ Other tool to facilitate patient care rounds
- ☐ No tools are used to facilitate patient care rounds

Rounding Instruments

26. You have indicated that in your ICU a rounding checklist, goal sheet, and/or tool is used. Could you please share this instrument to the researchers so that we may learn from it? Tools may be emailed to jkholodi@ucalgary.ca or faxed to 403-283-9994.

If you are not the best person to obtain a copy of the instrument from could you please provide the contact information below of the best person to obtain a copy of the tool below?

Outcomes of the Rounding Process

27. How often to medical errors occur as a result of rounding processes in your ICU?

- ◯ Rarely or Never
- ◯ Sometimes (< ½ of all patient rounded on)
- ◯ Usually (> ½ of all patients rounded on)
- ◯ Always

28. How efficient are patient care rounds in your ICU?

- ◯ Rounds are never efficient – time, ideas, and energy are always wasted
- ◯ Rounds are sometimes efficient – times, ideas, and energy are usually wasted
- ◯ Rounds are usually efficient – time, ideas and energy are sometimes wasted
- ◯ Rounds are always efficient – time, ideas, and energy are never wasted

29. How often do patient care rounds result in a tangible care plan for the patient in your ICU?

- ◯ Patient care rounds never result in a tangible care plan
- ◯ Patient care rounds sometimes result in a tangible care plan
- ◯ Patient care rounds usually result in a tangible care plan
- ◯ Patient care rounds always result in a tangible care plan

30. How often does the rounding process slow patient care in your ICU? For example, is the patient ever kept waiting for care (consults, discharge, tests/procedures) because they haven't been rounded on yet?

- ◯ Patient care is never slowed by patient care rounds
- ◯ Patient care is sometimes slowed by patient care rounds
- ◯ Patient care is usually slowed by patient care rounds
- ◯ Patient care is always slowed by patient care rounds

31. How often are patient care rounds equitable in your unit?

- ◯ Never (no patients receive time and attention they require)
- ◯ Sometimes (< ½ of patients receive the time and attention they require)
- ◯ Usually (> ½ of the patients receive the time and attention they require)
- ◯ Always (all patients receive the time and attention they require)

Satisfaction with the Rounding Process

32. On a scale from 1 through 10 please rate the quality of patient care rounds in your unit.

- ◯ 1 – worst possible rounds
- ◯ 2
- ◯ 3
- ◯ 4
- ◯ 5
- ◯ 6
- ◯ 7
- ◯ 8
- ◯ 9
- ◯ 10 – best possible rounds

33. On a scale from 1 through 10 please rate how much opportunity there is for improvement in patient care rounds in your unit.

- ◯ 1 – No possible room for improvement
- ◯ 2
- ◯ 3
- ◯ 4
- ◯ 5
- ◯ 6
- ◯ 7
- ◯ 8
- ◯ 9
- ◯ 10 – Great room for improvement

34. In your opinion, what are the best aspects of patient care rounds?

35. In your opinion, what are the worst aspects of patient care rounds?

36. Do you have any additional comments regarding patient care rounds?

National Survey of ICU Patient Care Rounds

Follow Up Interviews

37. We would like to briefly interview you to learn more about your experiences with patient care rounds. Can you provide the best e-mail or telephone number to reach you at?

38. If you are not the best person in your department to speak with regarding patient care rounds, could you please direct us to the person who would be more appropriate for us to contact?
