# Supplementary material for: A Survey of Rounding Practices in Canadian Adult Intensive Care Units
Source: PLoS One. 2015 Dec 23;10(12):e0145408. doi: 10.1371/journal.pone.0145408 (PMC4689549; doi:10.1371/journal.pone.0145408)
Supplement: S2 File — (PDF) [file pone.0145408.s002.pdf]

Semi-Structured Interview Questions

General Interview Questions

1. Briefly describe the purpose of patient care rounds and what should be accomplished during patient care rounds?

2. What are the best aspects of patient care rounds? Why is this the best aspect? Why does this work?

3. What are the worst aspects of patient care rounds? Why is this the worst aspect? Why doesn't this work?

4. How could patient care rounds be improved?

5. What roles do different participants of patient care rounds play?
    - prompts – patient, patient's family, bedside nurse, attending physician, trainees, respiratory therapists, pharmacist etc.
    - **Be sure to prompt based on answers given in survey.**

6. How is the patient's care plan ordered and documented?
    - prompts – notes taken by hand? computer? delegated note taker?

Semi-Structured Interview Questions

Tool Use Questions – these questions are only asked if the survey has indicated a tool is used during rounds

1. What is the purpose of the rounding tool(s) used in your ICU? What role do rounding tools play in rounds?

2. How was your rounding tool selected/created? How was the tool implemented? How is the tool used?

- prompts – are there any facilitators/barriers to the use?

3. Who benefits the most from the use of the tool?

4. How well does the tool work? What could be improved?

