# Supplementary material for: A Survey of Rounding Practices in Canadian Adult Intensive Care Units
Source: PLoS One. 2015 Dec 23;10(12):e0145408. doi: 10.1371/journal.pone.0145408 (PMC4689549; doi:10.1371/journal.pone.0145408)
Supplement: S3 File — Table A. Survey Responses. (PDF) [file pone.0145408.s003.pdf]

**Supplemental File 3**

Table A. Survey Responses

**Table A. Raw Survey Results**

| # | Question | Responses (N=111) |
|---|---|---|
| 1 | What is your role in the Intensive Care Unit | |
| | Medical Director | 45 |
| | ICU Physician | 49 |
| | Nurse Manager | 12 |
| | Patient Care Coordinator | 1 |
| 2 | *Responses contained identifying information* | |
| 3 | What province is your hospital located in? | |
| | Alberta | 13 |
| | British Columbia | 21 |
| | Manitoba | 4 |
| | New Brunswick | 3 |
| | Newfoundland | 1 |
| | Nova Scotia | 2 |
| | Ontario | 32 |
| | Prince Edward Island | 0 |
| | Quebec | 29 |
| | Saskatchewan | 6 |
| 4 | What types of patients are usually cared for in your | |
| | ICU?* | 110 |
| | Medical | 108 |
| | Surgical | 61 |
| | Cardiac – Medical | 56 |
| | Neurologic | 44 |
| | Trauma | 20 |
| | Cardiac – Surgical | 14 |
| | Burns | |
| 5 | Can your ICU care for ventilated patients? | |
| | Yes | 109 |
| | No | 1 |
| 6 | On, average how many operational beds are in your ICU? | Median: 13 (IQR 12)<br>Range: 3 – 38 |
| 7 | Does your ICU use an open or closed model of care? | |
| | Open | 15 |
| | Closed | 94 |
| | Mixed | 2 |
| 8 | Does your ICU regularly host residents? | |
| | Yes | 79 |
| | No | 32 |

| # | **Question** | **Responses (N=111)** |
|---|---|---|
| 9 | Which of the following types of rounds happen on a daily basis in your unit?* | |
| | Pre-rounding | 67 |
| | Multidisciplinary rounds | 89 |
| | Post-rounding | 52 |
| | Sign-out rounds | 70 |
| | Diagnostic imaging rounds | 31 |
| | Rapid fire or bullet rounds | 54 |
| 10 | In your unit who is involved in patient care rounds on a regular basis?* | |
| | Attending Physician | 108 |
| | ICU Fellows | 33 |
| | ICU Residents | 64 |
| | Medical Students | 47 |
| | Bedside Nurses | 103 |
| | Other Nursing Positions | 61 |
| | Nurse Practitioners | 14 |
| | Pharmacists | 94 |
| | Respiratory Therapists | 98 |
| | Dieticians | 81 |
| | Physiotherapists | 50 |
| | Social Workers | 41 |
| | Other | 7 |
| 11 | In your unit where do patient care rounds regularly occur?* | |
| | At the patients bedside | 59 |
| | In the hallway by the patients bed | 48 |
| | In a conference room | 13 |
| | At a nursing station | 6 |
| 12 | Is there a standard starting time for patient care rounds in your unit? | |
| | Never (0 days per week) | 11 |
| | Sometimes (1 – 3 days per week) | 11 |
| | Usually (4 – 6 days per week) | 49 |
| | Always (7 days per week) | 33 |
| 13 | Is there a standard starting location for patient care rounds in your unit? | |
| | Never (0 days per week) | 34 |
| | Sometimes (1 – 3 days per week) | 13 |

| # | Question | Responses (N=111) |
|---|---|---|
| | Usually (4 – 6 days per week) | 26 |
| | Always (7 days per week) | 33 |
| 14 | Are patient care rounds an open environment in your unit where all participants feel safe to voice their opinion? | |
| | Never | 1 |
| | Sometimes (< ½ of patients rounded on) | 5 |
| | Usually (> ½ of patients rounded on) | 35 |
| | Always | 64 |
| 15 | Are patient care rounds a collaborative environment in your unit where all health care providers are encouraged to participate and all opinions are considered when creating the patient care plan? | |
| | Never | 2 |
| | Sometimes (< ½ of patients rounded on) | 13 |
| | Usually (> ½ of patients rounded on) | 36 |
| | Always | 54 |
| 16 | How often are patient care rounds interrupted by answering pages? | |
| | Never | 10 |
| | Sometimes (< ½ of patients rounded on) | 633 |
| | Usually (> ½ of patients rounded on) | 21 |
| | Always | 10 |
| | How often are patient care rounds interrupted by answering phone calls? | |
| | Never | 6 |
| | Sometimes (< ½ of patients rounded on) | 64 |
| | Usually (> ½ of patients rounded on) | 26 |
| | Always | 9 |
| | How often are patient care rounds interrupted by requesting consultations? | |
| | Never | 21 |
| | Sometimes (< ½ of patients rounded on) | 68 |
| | Usually (> ½ of patients rounded on) | 10 |
| | Always | 2 |
| | How often are patient care rounds interrupted by receiving consultations? | |
| | Never | 21 |
| | Sometimes (< ½ of patients rounded on) | 68 |

| # | Question | Responses (N=111) |
|---|---|---|
| | Usually (> ½ of patients rounded on) | 14 |
| | Always | 2 |
| | How often are patient care rounds interrupted by emergent issues concerning other patients? | |
| | Never | 10 |
| | Sometimes (< ½ of patients rounded on) | 77 |
| | Usually (> ½ of patients rounded on) | 15 |
| | Always | 2 |
| | How often are patient care rounds interrupted by non-emergent issues concerning other patients? | |
| | Never | 34 |
| | Sometimes (< ½ of patients rounded on) | 55 |
| | Usually (> ½ of patients rounded on) | 15 |
| | Always | 1 |
| | How often are patient care rounds interrupted by code blues? | |
| | Never | 56 |
| | Sometimes (< ½ of patients rounded on) | 41 |
| | Usually (> ½ of patients rounded on) | 5 |
| | Always | 1 |
| | How often are patient care rounds interrupted by rapid response/medical emergency calls? | |
| | Never | 66 |
| | Sometimes (< ½ of patients rounded on) | 26 |
| | Usually (> ½ of patients rounded on) | 6 |
| | Always | 2 |
| | How often are patient care rounds interrupted by new patient admissions? | |
| | Never | 27 |
| | Sometimes (< ½ of patients rounded on) | 69 |
| | Usually (> ½ of patients rounded on) | 6 |
| | Always | 2 |
| | How often are patient care rounds interrupted by tests and/or procedures? | |
| | Never | 32 |
| | Sometimes (< ½ of patients rounded on) | 64 |
| | Usually (> ½ of patients rounded on) | 7 |
| | Always | 1 |

| # | Question | Responses (N=111) |
|---|---|---|
| 17 | If the patient is awake and aware, how is the patient handled during patient care rounds in your unit? | |
| | The patient is allowed and encouraged to attend rounds | 28 |
| | The patient is allowed but **not** encouraged to attend rounds | 32 |
| | The patient is allowed to attend but it is preferable that they do not | 28 |
| | The patient is not allowed to attend | 18 |
| 18 | If the patient is to participate in rounds what is their role?* | |
| | Observe | 46 |
| | Provide additional information | 60 |
| | Ask questions | 59 |
| | Participate in shared decision making | 38 |
| | Receive an update | 59 |
| 19 | How is the family handled during patient care rounds in your unit? | |
| | The family is allowed and encouraged to attend rounds | 12 |
| | The family is allowed but **not** encouraged to attend rounds | 32 |
| | The family is allowed to attend but it is preferable that they do not | 26 |
| | The family is not allowed to attend | 36 |
| 20 | If the family of the patient is to attend rounds, what is their role?* | |
| | Observe | 48 |
| | Provide additional information | 48 |
| | Ask questions | 50 |
| | Participate in shared decision making | 28 |
| | Receive an update | 48 |
| 21 | How often does the typical family of the patient attend patient care round? | |
| | Never (0 days per week) | 21 |
| | Sometimes (1 – 3 days per week) | 42 |
| | Usually (4 – 6 days per week) | 6 |
| | Always (7 days per week) | 0 |

| # | Question | Responses (N=111) |
|---|---|---|
| 22 | How many patients are typically rounded on in one day? | Median: 11 (IQR 6)<br>Range: 1 – 30 |
| 23 | How much time (in minutes) is typically spent with each patient | Median: 15 (IQR 10)<br>Range: 4 – 45 |
| 24 | What percentage of patient care rounds is spent on the following items: | |
| | Patient Care | Median: 80% (IQR 17.5%) |
| | Teaching | Median: 20% (IQR 15%) |
| | Other | Median: 0% (IQR 5%) |
| 25 | Does your unit use any of the following during patient care rounds:* | |
| | A rounding checklist | 31 |
| | A patient goal sheet | 16 |
| | Other tool to facilitate patient care rounds | 17 |
| | No tools are used to facilitate rounds | 53 |
| 27 | How often to medical errors occur as a result of rounding processes in your ICU? | |
| | Never | 58 |
| | Sometimes | 43 |
| | Usually | 0 |
| | Always | 0 |
| 28 | How efficient are patient care rounds in your ICU? | |
| | Never | |
| | Sometimes | 4 |
| | Usually | 17 |
| | Always | 70 |
| | | 11 |
| 29 | How often do patient care rounds result in a tangible care plan for the patient in your ICU? | |
| | Never | 0 |
| | Sometimes | 11 |
| | Usually | 63 |
| | Always | 29 |
| 30 | How often does the rounding process slow patient care in your ICU? | |
| | Never | 24 |
| | Sometimes | 74 |
| | Usually | 5 |
| | Always | 0 |

| # | **Question** | **Responses (N=111)** |
|---|---|---|
| 31 | How often are patient care rounds equitable in your unit?<br>Never<br>Sometimes<br>Usually<br>Always | <br><br>1<br>11<br>48<br>42 |
| 32 | On a scale from 1 through 10 please rate the quality of patient care rounds in your unit | Median: 7 (IQR 1)<br>Range: 3 – 10 |
| 33 | On a scale from 1 through 10 please rate how much opportunity there is for improvement in patient care rounds in your unit | Median: 7 (IQR 3)<br>Range: 2 – 10 |

*Numbers may sum to greater than 111 as multiple answers could be selected
