# Supplementary material for: A Survey of Rounding Practices in Canadian Adult Intensive Care Units
Source: PLoS One. 2015 Dec 23;10(12):e0145408. doi: 10.1371/journal.pone.0145408 (PMC4689549; doi:10.1371/journal.pone.0145408)
Supplement: S4 File — Figure A. Health Care Providers Who Regularly Attend Patient Care Rounds. Figure B. Evaluation of Rounds Using the IOM Aims for 21st Century Health Care[58]. Figure C. Relationship Between Self-Reported Rounding Quality and Room For Improvement Scores. Table A: Role of the Patient and Family During Rounds. Table B: Characteristics of Tools Provided. (PDF) [file pone.0145408.s004.pdf]

Supplemental File 4

Figure A. Health Care Providers Who Regularly Attend Patient Care Rounds

Figure B. Evaluation of Rounds Using the IOM Aims for 21st Century Health Care(1)

Figure C. Relationship Between Self-Reported Rounding Quality and Room For Improvement Scores

Table A: Role of the Patient and Family During Rounds

Table B: Characteristics of Tools Provided

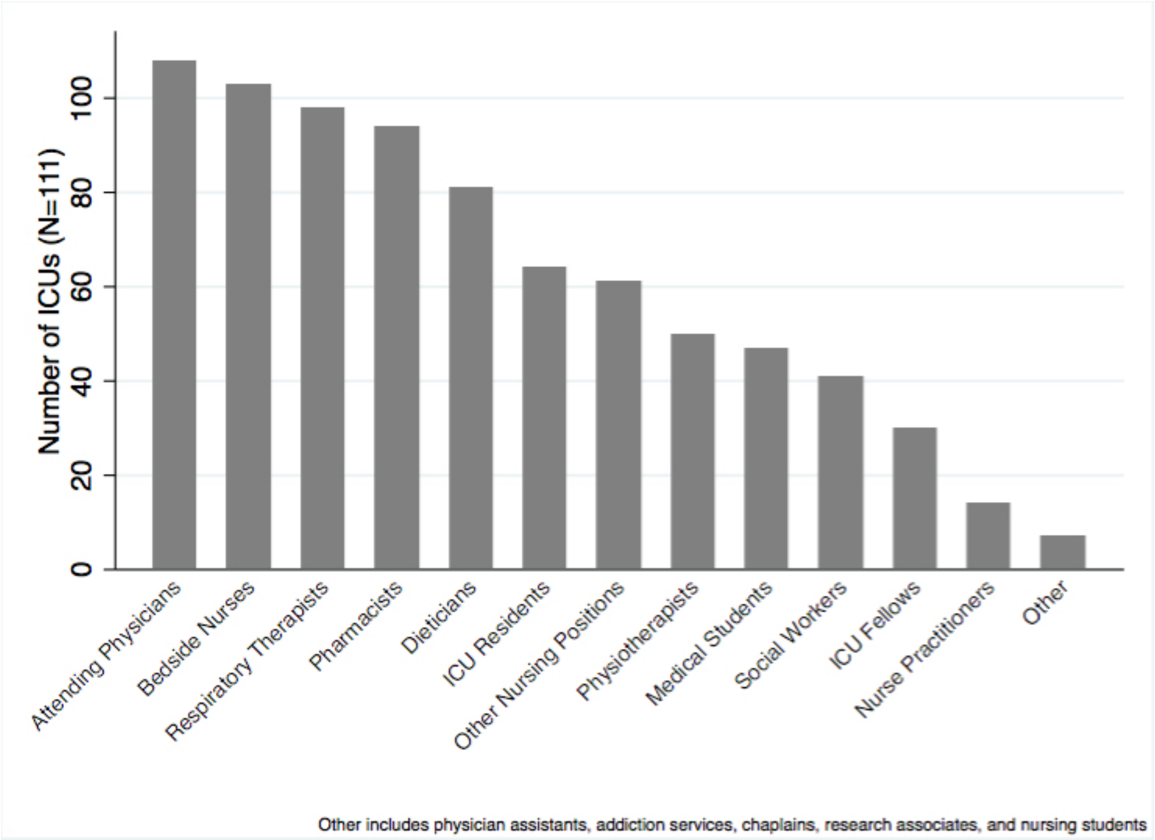

Figure A. Health Care Providers Who Regularly Attend Patient Care Rounds

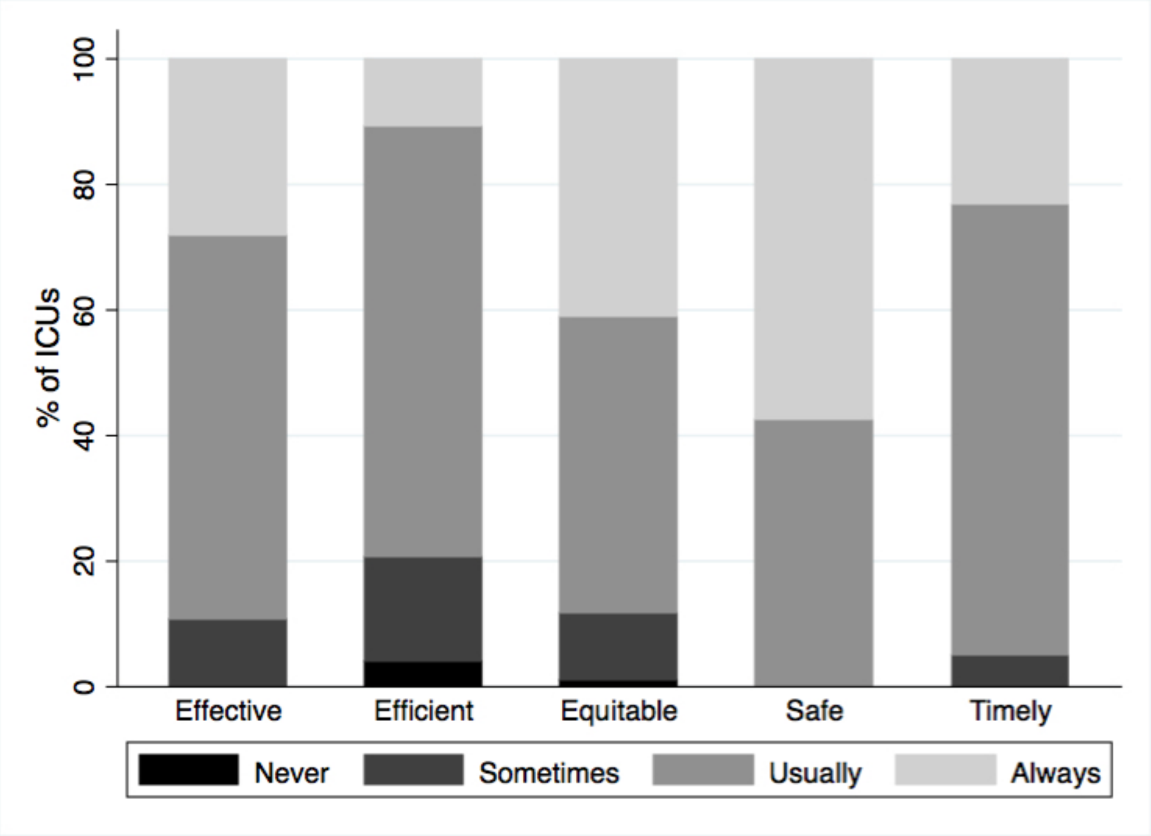

Figure B. Evaluation of Rounds Using the IOM Aims for 21st Century Health Care(1)

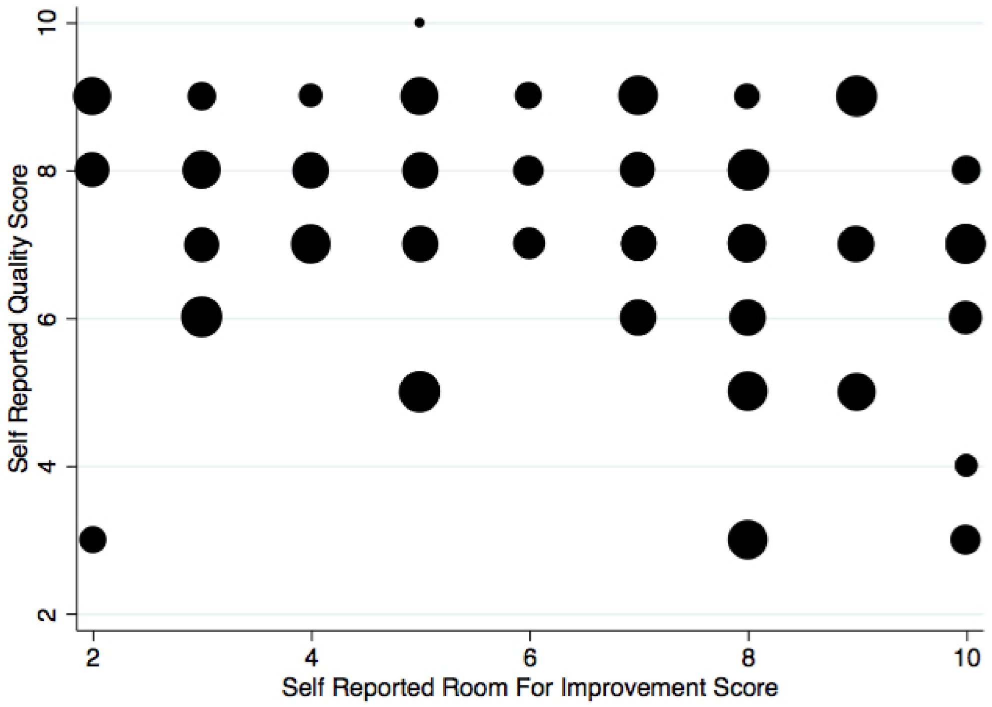

Each point represents an ICUs self reported quality score and self reported room for improvement score with the size of the data point reflecting the number of ICUs with that combination of scores. There was no association between self reported quality and room for improvement score (Chi-square test p-value = 0.2380).

Figure C. Relationship Between Self-Reported Rounding Quality and Room For Improvement Scores

Table A: Role of the Patient and Family During Rounds

Participant	Role (% of ICUs)[a]				
	Participate in Decision Making	Observe	Ask Questions	Receive an Update	Provide Information
Patient (N=92)	41%	50%	64%	64%	65%
Family (N=74)	38%	65%	68%	65%	65%

[a]Percentages sum to greater than 100% as patients and families were reported to have more than one role by some ICUs

Table B: Characteristics of Tools Provided

Characteristic	Checklist (N=9)	Worksheet (N=6)
Length		
Pages (range)	1 – 2	1 – 2
Number of Items		
Median (IQR)	16 (10 – 29)	42.5 (26 – 96)
Range	6 – 65	24 – 168
One Sheet Filled Out For:		
Each Individual Patient	7	6
All Patients	2	0
Designed To Be Written On:		
Yes	4	6
No	5	0
Internally Developed	7	6
Body System Focus		
One Body System	2	0
All Body Systems	7	6

References:

1. Institute of Medicine Committee on the Quality of Health Care in America: Crossing The Quality Chasm: A New Health System For The 21st Century. Washington, DC: National Academy Press; 2001.
