# Supplementary material for: A Survey of Rounding Practices in Canadian Adult Intensive Care Units
Source: PLoS One. 2015 Dec 23;10(12):e0145408. doi: 10.1371/journal.pone.0145408 (PMC4689549; doi:10.1371/journal.pone.0145408)
Supplement: S5 File — (PDF) [file pone.0145408.s005.pdf]

ICU Patient Care Rounds Guide

Date: ____________________

Affix Patient Label Here	**Diagnosis:**	
	**ICU Day:**	
	**Code Status:**	

	Reporting Items	Labs/Imaging (Abnormal Data Only)	Best Practices	Daily Goals/Plan
CNS	RASS<br>ICDSC/CAM-ICU<br>Sedation/Analgesia/Pain Score<br>GCS<br>Sleep<br>Seizures<br>ICP/CPP<br>CSF Drainage	Head CT/MRI:<br>Dilantin Level:<br>CSF:	Target RASS<br>Delirium Screening/Strategies<br>Sedation Vacation/SAT Candidate<br>Early Mobilization/Fall Risk<br>Restraints Required<br>Need for cEEG<br>Need for ICP Monitoring<br>Spinal Clearance	1.
CVS	BP<br>HR/Rhythm<br>CVP/Fluid Boluses<br>Max Temp<br>Vasopressors/Inotropes<br>Edema	K^+/Mg^{2+}:<br>CBC:<br>Echo:	DVT Prophylaxis<br>Leg Dopplers<br>Transfusion Trigger	2.
Respiratory	O_2 Mode/Requirements<br>Ventilator Mode/Settings<br>Respiratory Rate<br>O_2 Saturation<br>Nebulizers<br>Secretions<br>Chest Tubes	pH:<br>pCO_2/pO_2:<br>HCO_3/BE:<br>Chest Imaging:	VAP Prevention<br>SBT Candidate<br>Tracheostomy Candidate	
GI	Feed Type/Rate<br>Feed Tolerance<br>NG/OG placement/days in situ<br>Bowel Movements<br>Intra-abdominal Pressure<br>Wounds/Drains/Ostomies	Electrolytes:<br>Glucose:<br>LFT:	Early Nutrition<br>PUD Prophylaxis<br>Bowel Protocol<br>Glycemic Control	3.
GU	Urine Output<br>Fluid Balance (24h/Cumulative)<br>Foley placement/days in situ<br>Diuretics<br>Hemodialysis/CRRT	Creatinine/BUN:<br>Na^+/K^+:<br>HCO_3:	TFI Order<br>Fluid Balance Goal<br>Need for Dialysis	4.
IPC	Febrile<br>Antibiotics<br>Skin Integrity<br>Wounds<br>CVC site/days in situ<br>Arterial Line site/days in situ	WBC:<br>Cultures:<br>Abx Levels:	Isolation Protocol<br>Sepsis Protocol<br>Narrow/Stop Antibiotics<br>CLI Prevention	
Meds Labs	Current Medications/Dosages<br>Other Labs		Narrow/Stop Medications<br>Change Route<br>Adjust for Renal Function<br>Medication Reconciliation	5.
Social	Social History<br>Social Work/Spiritual Care<br>Changes to Code Status<br>Patient/Family Concerns		Representative Identified<br>Patient/Family Updated<br>Early Discharge Planning	
Research	Eligible for Research<br>On Research Protocol			
